# Negative Pressure Provides Simple and Stable Droplet Generation in a Flow-Focusing Microfluidic Device

**DOI:** 10.3390/mi12060662

**Published:** 2021-06-05

**Authors:** Nikita A. Filatov, Anatoly A. Evstrapov, Anton S. Bukatin

**Affiliations:** 1 Laboratory of Renewable Energy Sources, Alferov Saint Petersburg National Research Academic University of the Russian Academy of Sciences, 194021 Saint Petersburg, Russia; nikita.filatov@inbox.ru; 2 Laboratory of Bio and Chemosensor Microsystems, Institute for Analytical Instrumentation of RAS, 198095 Saint-Petersburg, Russia; an_evs@mail.ru

**Keywords:** droplet microfluidics, flow focusing, water-in-oil droplets, negative pressure, emulsion

## Abstract

Droplet microfluidics is an extremely useful and powerful tool for industrial, environmental, and biotechnological applications, due to advantages such as the small volume of reagents required, ultrahigh-throughput, precise control, and independent manipulations of each droplet. For the generation of monodisperse water-in-oil droplets, usually T-junction and flow-focusing microfluidic devices connected to syringe pumps or pressure controllers are used. Here, we investigated droplet-generation regimes in a flow-focusing microfluidic device induced by the negative pressure in the outlet reservoir, generated by a low-cost mini diaphragm vacuum pump. During the study, we compared two ways of adjusting the negative pressure using a compact electro-pneumatic regulator and a manual airflow control valve. The results showed that both types of regulators are suitable for the stable generation of monodisperse droplets for at least 4 h, with variations in diameter less than 1 µm. Droplet diameters at high levels of negative pressure were mainly determined by the hydrodynamic resistances of the inlet microchannels, although the absolute pressure value defined the generation frequency; however, the electro-pneumatic regulator is preferable and convenient for the accurate control of the pressure by an external electric signal, providing more stable pressure, and a wide range of droplet diameters and generation frequencies. The method of droplet generation suggested here is a simple, stable, reliable, and portable way of high-throughput production of relatively large volumes of monodisperse emulsions for biomedical applications.

## 1. Introduction

Nowadays, microfluidics is becoming a useful and popular tool in biological research and is finding its way into medical diagnostics. This has led to a significant growth in research publications in scientific journals [1,2,3] because microfluidics, combined with microelectronics and chemical engineering, offers new methods and technologies for creating lab-on-a-chip [4,5,6] and point-of-care devices [7,8,9], and high-throughput and highly sensitive screening systems [10]. Namely, injection, preparation, separation, detection, and other stages of an analysis can be integrated into one single microfluidic device. Such implementation of an assay requires small volumes of reagents and has lower thermal inertia; thus, mixing of reagents, heating, and cooling are carried out much faster [11,12,13]. This leads to an improvement in the sample and reagents dosing precision; thereby, the reproducibility of the assay and its speed increase, while the costs decrease. Moreover, an integration of the electrochemical, acoustofluidic, or optical sensing elements can increase the assay efficiency, reduce detection limits, and facilitate the construction of multiparameter devices that have a compact size and low price [14,15,16].

The most promising and fast-growing area of microfluidics is called droplet microfluidics. It operates with water-in-oil droplets, which serve as picoliter and nanoliter microreactors to perform biochemical reactions and assays. For high-throughput production of such monodisperse droplets with generation frequencies up to several kHz, T-injection and flow-focusing microfluidic devices are commonly used [17,18,19]. For complex assays, droplets can be controllably merged, divided, or injected with required reagents, and then sorted by fluorescent signals [20,21,22,23]. This approach can significantly increase the efficiency of drug screening, single-cell analysis, and other assays [24,25]. Moreover, droplet microfluidics provides single-molecule detection by droplet digital PCR [26], single-cell mRNA sequencing [27], and single-cell transcriptomic and proteomic analysis [28]. Other prospective applications of monodispersed emulsions include the synthesis of single-layer and multilayer microparticles with complex internal structure, and nanoparticles that can be used as containers for drug delivery, tissue engineering, and microlenses for photonics and biosensing applications [29,30,31,32,33].

Currently, there are several commercial setups that are based on droplet microfluidics techniques. Among them are the QX100/QX200 system for droplet digital polymerase chain reaction (Bio-Rad, Hercules, CA, USA); the Nadia instrument (microfluidic droplet-based platform for single cell research) by Dolomite Bio (Royston, UK); and the Chromium Single-Cell System by 10X Genomics (Pleasanton, CA, USA) for single-cell expression profiling technology, which allows high-throughput single-cell transcriptomics of many different cell types, as well as single-nuclei expression profiling [34]. Moreover, there are several companies, such as Elveflow (Paris, France), Fluigent (Jena, Germany), Dolomite-microfluidics (Royston, UK) that supply equipment, such as pressure controllers and syringe pumps, as well as microfluidic devices and consumables, for experimental protocols using droplet microfluidics. In these protocols, liquids are usually introduced into microfluidic devices, applying positive pressures to the inlets of the device using a pressure controller or syringe pumps [35]. However, the complex protocols require introduction of four and more liquids into the device [36], which requires at least four channel pressure controllers or syringe pumps, and precise tuning of all the components of the experimental setup. To overcome these limitations and simplify droplet generation, a method was proposed of introducing liquids into a microfluidic device under negative pressure, applied to the outlet reservoir manually by a simple syringe [37,38]. Moreover, a combination of positive and negative pressures applied to the device can provide additional functionality, such as the generation of droplets on demand [39]. For point-of-care applications, a special vacuum module can be integrated into the microfluidic droplet generator, so no external equipment is required to produce water-in-oil droplets [40]. Droplet generation induced by negative pressure in the outlet reservoir is thus a promising technique that can simplify the microfluidic setup and find wide applications in the fields of chemical synthesis, ultrahigh-throughput screening, and point-of-care testing; however, there are no affordable methods of controlling and changing the negative pressure, and little is known about how the pressure value and geometrical characteristics of microfluidic devices influence generation regimes.

In this work, we used a low-cost mini diaphragm vacuum pump to introduce liquids under negative pressure into a flow-focusing microfluidic device, and investigated water-in-oil droplet generation regimes in this setup. During the study, we compared two methods of adjusting the negative pressure: with a compact electro-pneumatic regulator and with a manual airflow pressure valve. In both cases, we analyzed the stability of droplet generation, and how droplet diameters and generation frequencies depended on applied pressure and on the hydrodynamic resistances of the inlet microchannels. The results showed that both methods of controlling negative pressure in the outlet reservoir are suitable for the production of monodispersed water-in-oil emulsions, and that the electro-pneumatic regulator is more suitable for precise experiments. Droplet diameters at high levels of negative pressure are mainly determined by the hydrodynamic resistances of the inlet channels, although their generation frequencies depend on the absolute value of the negative pressure, which is in agreement with a previous study [37].

## 2. Materials and Methods

**Figure 1:**
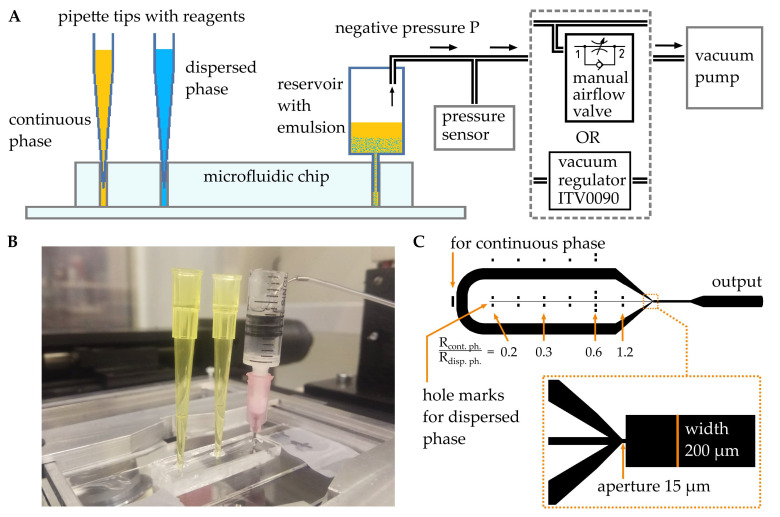
Droplet generation in a flow-focusing microfluidic device by applying negative pressure to the output reservoir: (**A**) circuit diagram of the setup; (**B**) general view of the microfluidic device; (**C**) design of the microfluidic chip.

The experimental setup consisted of a flow-focusing microfluidic chip with two inputs with inserted pipette tips (200 µL) containing the required reagents, one output collection reservoir, a regulator, and a vacuum pump (Figure 1A,B). To generate emulsion by negative pressure, we used a low-cost mini diaphragm vacuum pump (KVP8 PLUS-KJ-S, 24 V, 9 W, Kamoer Fluid Tech, Shanghai, China). A hand-operated unidirectional air flow control valve RFU 482-1/8 (Camozzi Automation spa, Brescia, Italy) or an electro-pneumatic regulator ITV0090-2L (SMC Corporation, Tokyo, Japan) was used to change the negative pressure value in the output reservoir, while the vacuum pump always worked at full capacity at the maximum voltage of 24 V. The flow control valve was used to dilute the airflow from the output reservoir with the atmospheric air, while the ITV0090 regulator directly controlled the pressure steplessly in proportion to an electric signal from an external voltage regulator (0–5 V) using a built-in electromagnetic valve, pressure sensor, and a feedback loop. The collection reservoir was composed of a typical fitting and a 5 mL buried plastic syringe. To monitor the negative pressure, a MPX2200DP pressure sensor (NXP Semiconductors, Eindhoven, Netherlands) was used. The data from it were smoothed in Origin Pro 2017 by the Loess smoothing method (span = 0.1).

The microfluidic chips with flow-focusing droplet generators (Figure 1C) were designed in Autodesk AutoCAD software. To investigate how the hydrodynamic resistances of the input microchannels influenced the droplet generation regimes, the chips contained long input channels with special marks. This design allowed us to create inlet holes at different distances from the droplet formation area and change the hydrodynamic resistance of the inlet microchannel for the dispersed phase.

The standard soft lithography technique [41,42] was used to fabricate microfluidic chips (Figure 1B) from polydimethylsiloxane (PDMS, Sylgard 184, Dow Silicones, Midland, MI, USA). Briefly, the mold was fabricated by contact optical lithography with a chromium mask on a silicon wafer with a 45 µm layer of SU–8 2025 (Kayaku Advanced Materials, Westborough, MA, USA). Then, the PDMS mix, containing prepolymer and the curing agent in a ratio of 10:1 *w*/*w*, was carefully mixed, degassed, and poured into the mold. After the curing step in an oven at 65 °C for 4 h, the PDMS replica was detached from the mold and cut onto separate devices. The inlets and outlets were fabricated using 1 mm biopsy puncher. To cover a PDMS replica with a glass slide, an oxygen plasma treatment was used. To create a hydrophobic coating on the inner walls of the microchannels and achieve a contact angle of ~100°, an anti-rain agent (Turtle Wax, Addison, IL, USA) was used. In the obtained devices, the microchannels height was 45 µm, the output channel width was 200 µm, and the aperture was 15 µm (Figure 1C). The overall dimensions of the device were chosen so that the distance from the microchannels to the boundaries of the PDMS was at least 4 mm to prevent air from entering the channels through the PDMS due to its high gas permeability [43,44,45]. Additionally, all the couplings and interconnections were covered with mineral oil.

To produce the water-in-oil monodispersed emulsions in the microfluidic chips, we used light mineral oil (M5310, Merc, Burlington, NJ, USA) with a 3.5% *w*/*w* surfactant (Abil EM 180, Evonik Operations GmbH, Essen, Germany) as a continuous phase. MilliQ deionized water (DI) was used as a dispersed phase. An optical microscope (Axiovert 200, Zeiss, Oberkochen, Germany) with a camera (Daheng Imaging, Beijing, China) was used for the visual control of the droplet-generation process and for measurements of the droplet diameters and generation frequencies. They were performed manually using the captured video frames in ImageJ software. If the measured values were larger than the microchannel’s height, then the shape of the droplets was approximated by an ellipsoid. Thus, equivalent diameter of the spherical droplets was calculated.

## 3. Results and Discussion

Droplet generation in microfluidics flow-focusing devices occurs in dripping or jetting regimes due to the instability of the interface between two immiscible liquids [18]. The main parameters that determine droplet generation are the capillary numbers of the dispersed and the continuous phases, which describe the ratio between the surface tension and viscous friction: Ca = ηV/γ, where V is average velocity (m/s), η is viscosity (Pa∙s), and γ is interfacial tension (N/m). The most common way to introduce liquids into microfluidic devices is to use syringe pumps, which provide the constant flow rate Q. Thus, the average velocity V = Q/S, where S is the channels cross-section. Another way to introduce liquids is to create a constant pressure difference between the inlets and outlets of the microfluidic device. Thus, the flow rate can be determined as Q = ∆P/R_h_ and average velocity V = ∆P/SR_h_, where ∆P is the pressure difference and *R_h_* is the hydrodynamic resistance of the microchannels. Hydrodynamic resistance depends on the dimensions of the channels and the liquid’s viscosity. For a rectangular microchannel with length *L*, width *w*, and depth *h*, filled with liquid with viscosity η, hydrodynamic resistance can be calculated using the following equation [46]:

(1) Rh=12ηLwh3(1−0.63hw), h<w.

Therefore, by changing the pressures applied to the microfluidic device or hydrodynamic resistances of the channels, it is possible to obtain different capillary numbers of continuous and dispersed phases and control droplet diameters and their generation frequency. According to the equation, the hydrodynamic resistance linearly depends on the fluid’s viscosity and the channel’s length. Therefore, to investigate how the hydrodynamic resistances of the inlet microchannels influence droplets parameters, we varied the length of the channel for the dispersed phase, creating inlet holes at different distances from the droplet generation area according to the marks, presented on Figure 1C. The ratio between the resistances of the continuous and dispersed phases R_con.ph._/R_disp.ph_ in our case was in the range from 0.23 to 1.2.

**Figure 2:**
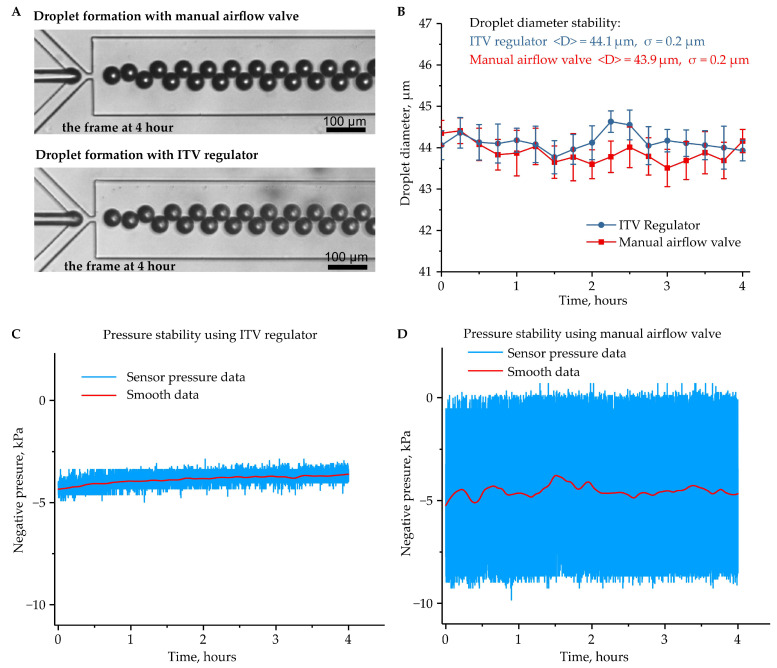
Stability of the droplet generation regimes. (**A**) Images of droplet generation in the microfluidics device using an ITV regulator and a manual airflow control valve; (**B**) droplet diameters during the 4 h stability test. Error bars indicate standard deviation. Pressure in the outlet reservoir was measured by an external pressure sensor during the 4 h stability test in the setup with the ITV regulator (**C**) and the manual airflow control valve (**D**). The experimental data were smoothed by Loess regression (span = 0.1).

To introduce liquids into the microfluidic device at different pressures, we used a mini diaphragm vacuum pump, which is a low-cost method of creating negative pressure in the outlet reservoir up to −95 kPa relative to atmospheric pressure. The main issue with this solution is the relatively large pressure variations in the vacuum line caused by diaphragm oscillations, which can significantly affect the droplet generation process and can lead to high variations in droplet parameters and finally to high polydispersity of the obtained water-in-oil emulsions. To reduce the influence of the diaphragm oscillations and to precisely control the pressure in the outlet reservoir, we compared two types of regulators: the ITV0090 electro-pneumatic regulator and the manual airflow control valve. For both types of regulators, we obtained stable generation of monodisperse water-in-oil emulsion in a dripping regime (Figure 2A). To define the stability of the generation, we measured the droplet diameters and the pressure in the outlet reservoir every 15 min for 4 h (Figure 2B). For every measurement in the setup with the ITV regulator and the manual valve, the coefficient of variation (CV) of the droplet diameter, which is defined as the ratio of the standard deviation to the mean value, was CV_ITV_ ≤ 1.2% or CV_valve_ ≤ 1.3%, respectively. Thus, the droplet generation was stable, and the water-in-oil emulsion collected in the outlet reservoir was monodisperse. For both types of regulators, the overall standard deviation of droplet diameter was about 0.2 µm, with an average diameter of 44 µm. The data from the pressure sensor showed a standard deviation of pressure in the outlet reservoir while using the ITV regulator, σ_ITV,_ of 0.3 kPa at an average pressure <P_ITV_> of −3.9 kPa (Figure 2C). While using the manual airflow control valve, the standard deviation was about 10 times higher: σ_valve_ = 2.9 kPa at an average pressure <P_valve_> = −4.5 kPa (Figure 2D). However, the high variability in pressure in the output reservoir did not affect the stability of the droplet generation regime while using the airflow control valve. Thus, both types of regulators can be used for the generation of monodispersed water-in-oil emulsions.

**Figure 3:**
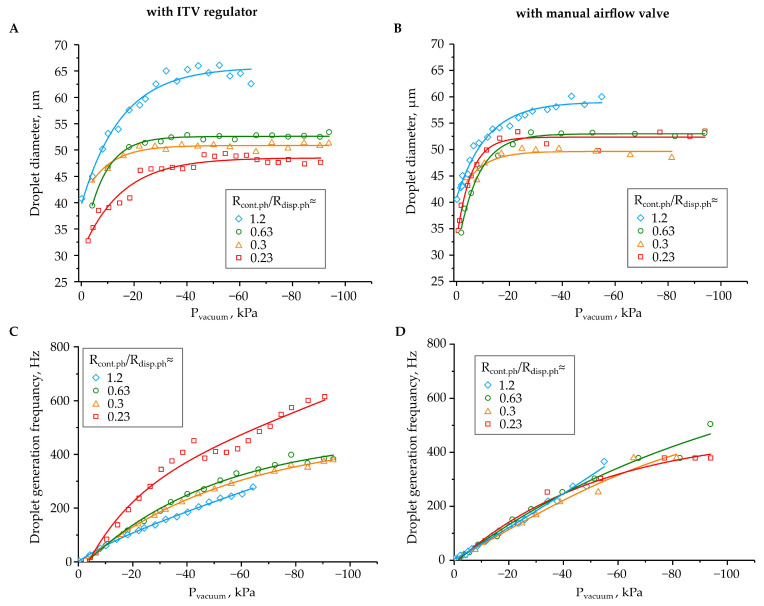
Characteristics of the droplet generation regimes in the flow-focusing microfluidic device at different negative pressures in the outlet reservoir, and different hydrodynamic resistances of inlet channels in the setup with the ITV pressure regulator or with the manual airflow control valve: (**A**,**B**) diameters of obtained droplets; (**C**,**D**) droplet generation frequencies. Colored lines are eye guides.

The experimental data presented in Figure 3 indicate that droplet diameters and their generation frequencies strongly depend on the value of the applied negative pressure in the outlet reservoir. As the pressure difference between the inlets and the outlet of the microfluidic device increased, liquid flow rates increased, which led to increases in droplet diameters, from 33–35 μm to 45–65 μm, depending on the hydrodynamic resistance ratio R_con.ph._/R_disp.ph_, while using the ITV regulator. However, at highly negative pressures, below −30 kPa, it was almost independent of the pressure. This happened due to droplet diameters being mostly determined by the ratio of the capillary numbers or flow rates of the dispersed and continuous phases, and is independent of their absolute values (Figure 3A,B) [18]. While using the airflow control valve, the droplet diameter range was slightly lower, from 35 μm to 60 μm.

Unlike the droplets’ diameters, their generation frequency monotonously increased with increasing negative pressure value in the outlet reservoir. In cases of droplet generation using the ITV regulator, the frequency range was from 0 Hz to 400–600 Hz, depending on the hydrodynamic resistances of the inlet channels (Figure 3C). Using the manual airflow control, the frequency range was slightly lower, from 0 Hz to 400–500 Hz, but was almost independent of the hydrodynamic resistances of the inlet channels (Figure 3D). We suppose that these differences in the obtained generation parameters are due to the pressure variations in the outlet reservoir, caused by the pump’s membrane oscillations, which were much lower when using the ITV regulator instead of the manual valve.

**Figure 4:**
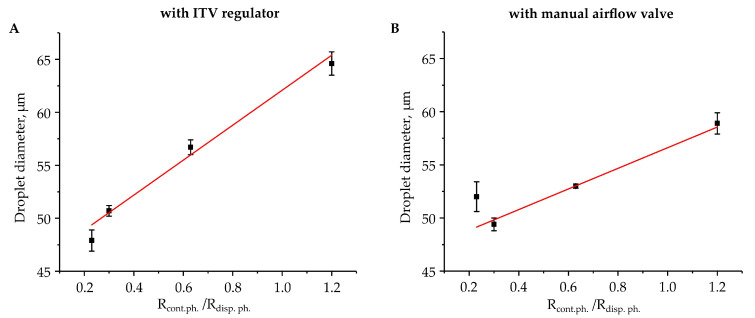
Droplets diameters as a function of the ratio between the hydrodynamic resistances of the continuous and the dispersed phases, R_con.ph._/R_disp.ph_. Droplet generation was performed in the setup with (**A**) the ITV regulator and (**B**) the manual airflow control valve. The pressure in the outlet reservoir was set to −50 kPa, when droplets diameter was insensitive to its value. Error bars indicate standard deviation.

Our further studies showed that at high levels of negative pressure, when droplets diameter did not depend on the pressure, it linearly depended on the ratio between the hydrodynamic resistances of the inlet channels R_con.ph._/R_disp.ph_ using either the ITV regulator or the manual airflow control valve (Figure 4). This relation indicates that droplet generation at high values of negative pressure in the outlet reservoir is insensitive to the variations in the pressure, is very stable, and can produce large volumes of monodisperse water-in-oil emulsions with droplets of required diameter. The hydrodynamic resistances of the inlet microchannels, which can be adjusted by changing their lengths or the viscosity of the continuous and dispersed phases, define the droplets’ diameters. For their accurate definition, several experimental tests should be performed. During these tests, microfluidics devices with the same droplet formation area and outlet channel, but with different lengths of inlet channels should be studied. Another method is to determine the flow rates using a single device and syringe pumps, or a multichannel pressure controller, and then calculate the required values of the hydrodynamic resistances. Alternatively, there are several approaches for qualitative and quantitative numerical simulations of the droplet formation in microfluidic devices, which can be used to adjust the hydrodynamic resistances of the inlet microchannels to obtain droplets with requested diameter [19,47,48].

## 4. Conclusions

In this work, we investigated monodisperse water-in-oil droplet generation regimes in a flow-focusing microfluidics device induced by the negative pressure in the outlet reservoir, while the inlet reservoirs were set to the atmospheric pressure. Here, we proposed to use a low-cost mini diaphragm vacuum pump and either an electro-pneumatic regulator SMC ITV0090-2L or a manual airflow control valve to generate and adjust the pressure in the reservoir.

Our experimental results showed that with both types of pressure regulators, the generation of a water-in-oil emulsion was stable for at least 4 h. The coefficient of variation of the droplet diameter in both cases was ≤1.3% and its standard deviation was 0.2 µm, while the average diameter was 44 µm. However, the ITV regulator suppressed the pressure variations in the outlet reservoir, caused by the pump membrane oscillations, which led to a slightly larger range of droplet diameters and generation frequencies. With the manual airflow regulator, the pressure variations were much bigger, though it did not influence droplet diameter.

Our further investigations indicated that at relatively high levels of negative pressure, below −30 kPa, droplet diameter does not depend on the absolute value of the pressure, but is determined by the ratio between the hydrodynamic resistances of the inlet microchannels. These resistances determine the flow rates and the capillary numbers of the continuous and dispersed phases. Therefore, droplet diameter linearly depends on the ratio between these hydrodynamic resistances. On the contrary, droplet generation frequency is determined by the total absolute values of the flow rates of both phases and monotonically depends on the applied negative pressure, showing weak dependence on the hydrodynamic pressures of the inlet microchannels.

Our studies showed that introducing liquids into a flow-focusing microfluidics device under a negative pressure is a convenient and reliable way of generating monodisperse water-in-oil emulsions with predefined droplet diameters. Both types of investigated pressure regulators are suitable for this purpose and provide similar results. However, the ITV series electro-pneumatic regulator is preferable and convenient for accurate control of the pressure by external electric signal compared with a manual airflow control valve. It does not require external pressure sensors and special calibration for obtaining reproducible results. To accurately determine the values of hydrodynamic resistances of the inlet microchannels for the specific types of liquids, several experimental tests or numerical simulations should be performed. After adjusting these values, droplet generation, induced by negative pressure in the outlet reservoir, is stable and insensitive to pressure variations; therefore, it can be used to generate large amounts of monodisperse emulsions and emulsions with complex internal structures, such as double and multilayer emulsions that require several types of continuous and dispersed phases. We think that the proposed and investigated method of droplet generation will be useful for the microfluidic community for development of new applications, lab-on-a-chip and point-of-care devices based on droplet microfluidics technologies.

## Data Availability

The data presented in this study are available on request from the corresponding author.
